# A Single-IMU Wearable System with 1D U-Net for Knee Adduction Moment Waveform Reconstruction During Gait

**DOI:** 10.3390/s26144421

**Published:** 2026-07-12

**Authors:** Hiroko Sakamoto, Hiroshi Yoshihara, Ayako Akiba, Kohei Nishizawa, Masaki Nagashima, Kengo Harato, Takeo Nagura, Masaya Nakamura

**Affiliations:** 1 Department of Orthopaedic Surgery, Keio University School of Medicine, 35 Shinanomachi, Shinjuku-ku, Tokyo 160-8582, Japan; ayako.ortho@keio.jp (A.A.); nishizawakohei32@gmail.com (K.N.); masa@keio.jp (M.N.); 2 Department of Health Economics and Outcomes Research, Graduate School of Pharmaceutical Sciences, The University of Tokyo, 7-3-1 Hongo, Bunkyo-ku, Tokyo 113-0033, Japan; no14nec0@gmail.com; 3 Department of Orthopaedic Surgery, International University of Health and Welfare Mita Hospital, 1-4-3 Mita, Minato-ku, Tokyo 108-8329, Japan; masakinagashima@hotmail.com; 4 Sports Medicine Research Center, Keio University, 4-1-1 Hiyoshi, Kohoku-ku, Yokohama, Kanagawa 223-8521, Japan; harato@keio.jp; 5 Department of Clinical Biomechanics, Keio University School of Medicine, 35 Shinanomachi, Shinjuku-ku, Tokyo 160-8582, Japan; nagura@keio.jp

**Keywords:** knee adduction moment, inertial measurement unit, wearable sensor, 1D U-Net, waveform reconstruction, gait analysis, knee osteoarthritis, biomechanical assessment, independent holdout validation, machine learning

## Abstract

**Highlights:**

**What are the main findings?**

- A single tibial IMU with a 1D U-Net reconstructed knee adduction moment waveforms during gait.
- The waveform-derived peak KAM and KAM impulse showed positive associations with reference motion-analysis-derived values.

**What are the implications of the main findings?**

- The system enables a clinically feasible assessment of medial knee loading without motion capture and force plates.
- Time-continuous waveform reconstruction enables evaluation of temporal KAM patterns and clinically relevant summary measures using a minimal wearable setup.

**Abstract:**

The knee adduction moment (KAM) is an important biomechanical marker of medial knee joint loading, but conventional assessment requires laboratory-based motion analysis. This study aimed to develop and evaluate an improved inertial measurement unit (IMU)-based KAM estimation system that incorporates a one-dimensional U-Net (1D U-Net) with a gated recurrent unit (GRU) bottleneck. Gait data from 50 participants were used for model development and internal evaluation, and an independent holdout dataset collected using the same study protocol included 45 participants. Simultaneously recorded IMU signals and reference KAM obtained from motion capture-based inverse dynamics were used for training and evaluation. The model used a single IMU attached to the tibial tuberosity to reconstruct the KAM waveform during the stance phase. For peak KAM, Pearson’s r values were 0.819, 0.602, and 0.621 for the training, internal validation, and holdout datasets, respectively. For KAM impulse, the corresponding values were 0.941, 0.744, and 0.745. These findings indicate that a simplified wearable system combining a single tibial IMU with a 1D U-Net incorporating a GRU bottleneck can reconstruct the KAM waveform during walking and derive clinically relevant parameters from it. Although further improvement in prediction accuracy is required, this framework may provide a practical basis for simplified screening or monitoring of medial knee loading in settings closer to routine clinical practice.

## 1. Introduction

Knee osteoarthritis (KOA) is a major cause of pain and functional disability and is widely recognized as a global public health challenge [1,2]. Numerous risk factors, including aging, obesity, trauma, and genetic predisposition, contribute to the onset and progression of KOA [3,4]. In addition to these systemic and structural factors, biomechanical factors play an important role in disease onset and progression [5].

Among biomechanical factors, dynamic frontal plane loading during gait is a key determinant of medial knee joint loading. Representative frontal plane loading features include varus thrust during the stance phase and knee adduction moment (KAM). Varus thrust has been associated with disease progression [6,7], whereas KAM, calculated using inverse dynamics based on three-dimensional motion analysis and force plate data, is widely accepted as a quantitative surrogate measure of medial knee joint loading [8,9,10,11]. Furthermore, increased KAM has been reported to predict structural progression of KOA [12,13,14], underscoring its clinical importance as a biomechanical marker.

Despite its importance, conventional KAM assessment requires a laboratory-based motion analysis system equipped with optical motion capture and force plates. These systems are expensive, require substantial space, and demand specialized technical expertise, which limits their accessibility and clinical applicability. Consequently, translating KAM assessment into routine clinical practice or real-world settings remains challenging. In response to these limitations, recent advances in wearable sensor technology have made it increasingly feasible to analyze gait outside the laboratory [15]. In particular, inertial measurement units (IMUs) have attracted considerable attention because they are compact, low-cost, and capable of capturing movement data in real time [16]. More broadly, wearable inertial sensors have been increasingly used for gait assessment in osteoarthritis and other musculoskeletal disorders [17]. In addition, recent studies have combined inertial sensor-derived gait features with machine learning to classify pathological gait patterns in neurological and orthopaedic populations, supporting the broader clinical potential of wearable sensor-based gait analysis [18,19].

However, many existing IMU-based KAM estimation systems still require multiple sensors and rely on complex calibration procedures or laboratory-derived data [20,21,22,23,24,25,26]. In addition, many previous models have focused primarily on estimating scalar outcomes such as peak KAM, rather than reconstructing the temporal structure of the KAM waveform over the entire stance phase [24,27,28]. Conversely, even among studies that have attempted waveform reconstruction, some have provided insufficient evaluations of clinically important waveform-derived parameters, such as the peak KAM and KAM impulse [29].

Therefore, a KAM estimation system intended for clinical application should be able to reconstruct the temporal structure of the KAM waveform itself using a minimal sensor configuration and consistently derive clinically important parameters from that waveform. Its performance should also be evaluated using independent holdout data.

In our previous work, we developed and reported a wearable system that estimates KAM during gait using a single six-axis IMU, demonstrating the potential for simple and clinically applicable assessment [30,31]. However, this earlier system was based on a convolutional neural network (CNN) model and was designed primarily to estimate scalar KAM metrics. Consequently, it had limitations in consistently reconstructing the temporal structure of the entire KAM waveform.

To address this limitation, we updated the previous CNN-based system by incorporating a one-dimensional U-Net (1D U-Net) with a gated recurrent unit (GRU) bottleneck to reconstruct the full KAM waveform rather than directly estimating individual parameters. The 1D U-Net uses an encoder–decoder architecture that is well suited for time-series reconstruction and waveform modeling, enabling temporal-resolution preservation while integrating multiscale features through skip connections [32,33]. In addition, a GRU bottleneck was incorporated to model the temporal dependencies within the compressed gait-signal representation. Peak KAM and KAM impulse were then derived from the reconstructed waveform. Model performance was evaluated in the training, internal validation, and independent holdout datasets.

This study aimed to develop and evaluate an improved single-IMU-based KAM estimation system incorporating a 1D U-Net with a GRU bottleneck. We hypothesized that this updated model would enable KAM waveform reconstruction and improve waveform-derived parameter estimation compared with our previous CNN-based model. Ultimately, this system is intended to support wearable biomechanical assessment in patients with KOA and contribute to the development of clinically deployable evaluation methods in settings closer to routine clinical practice and daily life.

## 2. Materials and Methods

### 2.1. Participants

This cross-sectional study included individuals who underwent gait assessment at Keio University Hospital and International University of Health and Welfare Mita Hospital between July 2023 and June 2024, comprising both healthy controls and patients with KOA. KOA severity was assessed using the Kellgren–Lawrence (KL) grading system [34]. Individuals who did not meet the diagnostic criteria for early KOA [35] and were graded as KL 0 on plain radiographs were from healthy controls. Therefore, all knees classified as KL 0 in this study were from healthy controls, whereas all KOA knees were classified as KL grade 1 or higher.

Gait data from 50 participants were used for model development and internal evaluation. The data were randomly split at the participant level into a training dataset of 40 participants (78 knees, 837 gait trials) and an internal validation dataset of 10 participants (17 knees, 114 gait trials). This participant-level split ensured that data from the same individual were not included in more than one dataset. To further assess model performance on unseen data, an independent holdout dataset was included. This dataset consisted of an independent cohort of 45 participants (88 knees, 281 gait trials) collected separately using the same study protocol. None of these data were used for training or internal evaluation.

Analyses were performed at the knee level. Because two knees and multiple gait trials were obtained from some participants, observations were not fully independent. Accordingly, data were split at the participant level, and all 95% confidence intervals were computed using a participant-level cluster bootstrap. Participant characteristics and distribution of KL grades in each dataset are summarized in Table 1.

The inclusion criterion was the ability to walk independently and complete the gait assessment protocol. The exclusion criteria were hip or ankle symptoms, prior total knee arthroplasty, and neurological or musculoskeletal disorders affecting gait, including rheumatoid arthritis and lumbar spinal stenosis.

### 2.2. Ethical Approval

This cross-sectional study was conducted in accordance with the Declaration of Helsinki. The study protocol was approved by the Institutional Review Board of Keio University School of Medicine (approval no. 20190294; 24 March 2020). Written informed consent was obtained from all participants prior to the study.

### 2.3. Gait Measurement Protocol

**Figure 1:**
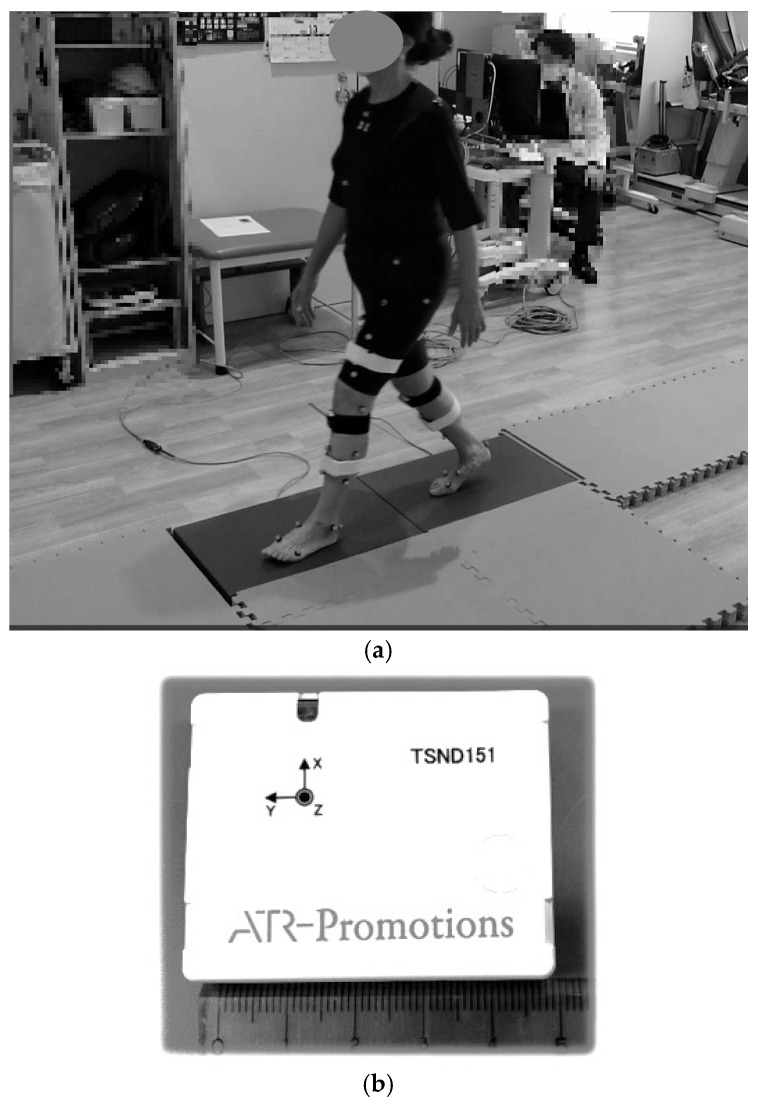
Experimental setup for simultaneous gait measurement using a motion capture system, force plates, and inertial measurement units (IMUs). (**a**) Gait measurement in the laboratory using a motion capture system and force plates, with IMU sensors attached to the bilateral tibial tuberosities.; (**b**) IMU sensor used in this study.

Gait measurements were performed in gait laboratory settings using three-dimensional motion capture systems and force plates. At the same time, IMUs were attached bilaterally over the tibial tuberosities to collect input data for the machine learning model (Figure 1). Commercially available IMUs (TSND151; ATR-Promotions, Kyoto, Japan) were used. One sensor was secured to each tibial tuberosity with a strap, and the sensor’s anterior–posterior axis was aligned with the participant’s forward direction. IMU signals, including triaxial acceleration and triaxial angular velocity, were sampled at 200 Hz and recorded using dedicated software (Sensor Controller; ATR-Promotions, Kyoto, Japan). Three-dimensional kinematic data were collected at 200 Hz using eight-camera motion capture systems (Oqus, Qualisys, Göteborg, Sweden, or Vicon MX, Vicon Motion System, Oxford, UK), while ground reaction forces were recorded synchronously at 2000 Hz using two force plates (AM6110, Bertec, Columbus, OH, USA, or AMTI OR6, Advance medical technology, Water town, MA, USA). Reflective marker trajectories were recorded using Qualisys Track Manager software (Version 2.7; Qualisys AB, Göteborg, Sweden) or Nexus software (Version 2.10; Vicon Motion System, Oxford, UK). After several practice trials, the participants walked along a 5 m walkway in the gait laboratory at a self-selected speed. For each participant, 6–10 walking trials were recorded. During post-processing, IMU data were temporally aligned with the motion capture data using gait events identified from the ground reaction force data.

Knee joint kinematics and kinetics were analyzed using Visual3D software (Version 6.00.19; C-Motion, Rockville, MD, USA). KAM was calculated using inverse dynamics from motion capture and ground reaction force data. The first peak during stance was defined as peak KAM, and KAM impulse was calculated as the time integral of the KAM throughout the stance phase [36]. KAM values were normalized to body weight and height and expressed as N·m/(BW·Ht).

### 2.4. Signal Processing

Prior to model training, the IMU signals were preprocessed to reduce noise and standardize the input data. First, the raw IMU signals were filtered using a zero-phase fourth-order Butterworth bandpass filter with cutoff frequencies of 1–16 Hz. A 1 Hz high-pass cutoff was used to remove baseline drift and gravitational/DC component, and a 16 Hz low-pass cutoff was selected to retain gait-related dynamics, including stride frequency components and their harmonics as well as heel-strike impact transients, while suppressing high-frequency sensor noise. A fourth-order Butterworth filter was used to provide a maximally flat passband with adequate roll-off, and zero-phase forward–backward filtering was applied to avoid phase distortion and waveform time shifts that could affect peak timing. This frequency range is consistent with previous reports [24,37].

The IMU sensor provided six signal channels consisting of triaxial acceleration and triaxial angular velocity. Of these, the x-axis angular velocity channel was excluded from the input feature set. In a channel ablation analysis performed under identical training conditions, including the x-axis angular velocity did not improve prediction accuracy and tended to reduce performance in the internal validation dataset (Supplementary Table S1). Accordingly, the x-axis angular velocity channel was used for gait-event detection during preprocessing but was excluded from the model input. Thus, five signal channels were used as inputs to the neural network.

In addition, to avoid boundary effects during convolution, 50 frames of padding were added to both ends of each input sequence. This procedure enabled the model to process the entire stance phase appropriately while preserving the temporal structure of the signals.

### 2.5. 1D U-Net Model Architecture

**Figure 2:**
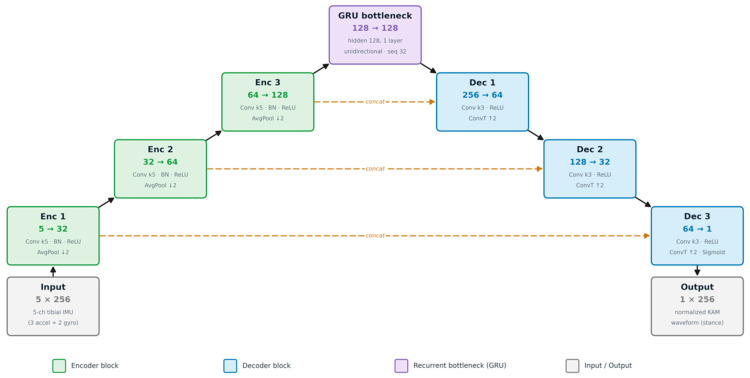
Architecture of the proposed 1D U-Net with a recurrent (GRU) bottleneck for reconstructing the KAM waveform from a single tibial IMU. The input is a five-channel IMU sequence consisting of three accelerometer and two gyroscope axes (5 × 256), and the output is a one-channel normalized KAM waveform over the stance phase (1 × 256). Each encoder block consists of a 1D convolution with a kernel size of 5, batch normalization, ReLU activation, and average pooling with a downsampling factor of 2, increasing the channel dimension at each stage (5 → 32 → 64 → 128). The bottleneck is a single-layer, unidirectional GRU with a hidden size of 128, applied to the length-32 feature sequence. Each decoder block consists of a 1D convolution with a kernel size of 3, ReLU activation, and a transposed convolution for upsampling by a factor of 2, with a final sigmoid activation in the last block mapping the output to the normalized range. Bold values indicate the input-to-output channel dimensions of each block. Solid arrows denote data flow, and dashed arrows denote skip connections that concatenate encoder feature maps with the corresponding decoder input channel-wise. Enc and Dec denote encoder and decoder blocks, respectively. The symbols “↓2” and “↑2” indicate downsampling and upsampling by a factor of 2, respectively.

To estimate the KAM waveform from IMU signals, we implemented a 1D U-Net-based encoder–decoder network with a recurrent bottleneck. The model consisted of three encoder blocks, a gated recurrent unit (GRU) layer at the bottleneck, and three decoder blocks, with skip connections between the corresponding encoder and decoder stages (Figure 2). The input to the model was a five-channel IMU time-series sequence resampled to 256 samples, and the output was a single-channel normalized KAM waveform corresponding to the stance phase.

In the encoder, temporal features were extracted hierarchically from the input IMU time-series signals through successive one-dimensional convolution and downsampling operations. Each encoder block consisted of a one-dimensional convolution, batch normalization, ReLU activation, dropout, and average pooling. The convolutional layers in the encoder used a kernel size of 5 with zero padding of 2. The numbers of output channels in the three encoder blocks were 32, 64, and 128. Average pooling with a kernel size of 2 and stride of 2 was used for downsampling, reducing the temporal length from 256 to 128, 64, and 32 samples across the encoder blocks. At each stage, the temporal resolution was reduced by a factor of two while higher-level feature representations were learned.

A GRU layer was placed at the bottleneck to capture temporal dependencies in the compressed feature sequence. The bottleneck consisted of a single-layer GRU with an input size and hidden size of 128, which processed the 32-step and 128-channel feature sequence.

In the decoder, the temporal resolution was progressively restored by upsampling, and high-resolution features from the corresponding encoder stages were integrated through skip connections to reconstruct the output waveform. Each decoder block concatenated the upsampled decoder feature map with the corresponding encoder feature map via a skip connection and then applied a one-dimensional convolution with a kernel size of 3 and padding of 1, ReLU activation, dropout, and a transposed one-dimensional convolution with a kernel size of 2 and stride of 2 for two-fold upsampling. The decoder channel widths mirrored the encoder in reverse order. The final transposed convolution produced a single-channel output, followed by a sigmoid activation function. This architecture enabled the model to capture both local temporal patterns and broader temporal context in the gait signals.

Padded sequences were used as inputs to the network to preserve the temporal structure and reduce boundary artifacts during convolution. The output waveform has the same temporal resolution as the input sequence. A dropout rate of 0.1 was employed in all convolutional blocks.

The proposed network contained approximately 3.0 × 10^5^ trainable parameters, providing sufficient representational capacity for biomechanical waveform reconstruction while maintaining computational efficiency.

### 2.6. Model Training

#### 2.6.1. Training Configuration

A multitask learning strategy was adopted for model training. The mean squared error (MSE) was minimized for peak KAM and KAM impulse values derived from the predicted and reference waveforms. In parallel, the shape difference between the predicted and reference waveforms was minimized using a soft-DTW loss [38]. The total loss was defined as a weighted composite loss combining the waveform-level soft-DTW loss and the scalar losses for peak KAM and KAM impulse as follows:

L = (2 × L_softDTW + L_peak + L_impulse)/4,(1)

where L_softDTW is the soft-DTW loss between the predicted and reference KAM waveforms with γ = 0.1, and L_peak and L_impulse are the MSE terms for the waveform-derived peak KAM and KAM impulse, respectively. Thus, the relative weights for the waveform, peak KAM, and KAM impulse terms were 2:1:1.

Optimization was performed using the AdamW optimizer with an initial learning rate of 2 × 10^−4^ and weight decay of 1 × 10^−5^. A cosine-annealing learning rate schedule with warm restarts was used, with T_0_ = 10 and η_min = 1 × 10^−6^. Training was conducted using mini-batches with a batch size of 64, and the maximum number of epochs was set to 200. The model was implemented in Python (Version 3.10) using PyTorch (Version 2.5.1; CUDA 12.1) and trained on a workstation equipped with an NVIDIA RTX 6000 Ada GPU. Mixed-precision training was enabled using automatic mixed precision.

#### 2.6.2. Overfitting Prevention and Model Selection

To prevent overfitting, early stopping was applied during training. The dataset was partitioned at the participant level into a training set, an internal validation set, and an independent holdout set, with no participant overlap across sets. The internal validation set was used to monitor training, and model and training choices were guided by internal validation performance. The independent holdout set was not used at any stage of training or model development and was reserved for final evaluation only. No separate validation subset was drawn from the training set. Specifically, training was terminated when the internal validation soft-DTW loss did not improve for 50 consecutive epochs. In addition, dropout layers were incorporated into the network to improve generalization by randomly deactivating a subset of neurons during training. The dropout rate was set to 0.1 in all convolutional blocks. As a data augmentation strategy, Gaussian noise with a signal-to-noise ratio ranging from 5.0 to 20.0 dB was randomly added to the input signals. The model with the lowest internal validation soft-DTW loss during training was selected as the final model. The random seed was set to 2022 to improve reproducibility.

### 2.7. Model Evaluation and Statistical Analysis

The performance of the proposed model was evaluated by comparing the IMU-estimated KAM with the reference KAM obtained using motion-capture-based inverse dynamics. Scalar performance of peak KAM and KAM impulse was evaluated using mean absolute error (MAE), root-mean-square error (RMSE; same units as KAM), mean absolute percentage error (MAPE; scale-independent relative error), Pearson correlation coefficient (linear association), Spearman’s ρ (rank association), and intraclass correlation coefficient (ICC (2,1); two-way random-effects model, absolute agreement). Agreement was further assessed using Bland–Altman analysis, including bias and 95% limits of agreement. Waveform reconstruction was quantified using range-normalized RMSE and dynamic time warping (DTW) distance between the predicted and reference KAM waveforms. Because both knees and multiple gait trials were obtained from some participant, 95% confidence intervals were estimated using a participant-level cluster bootstrap. To assess the robustness of the data split, participant-level 5-fold cross-validation was additionally performed using GroupKFold with grouping by participant, with no participant overlap between the training and test sets within each fold. The absolute error distributions between the internal validation and holdout datasets were compared using the Mann–Whitney U test. Because correlation quantifies association rather than agreement, ICC and Bland–Altman analyses were reported along with correlation coefficients. All analyses were performed using Python.

### 2.8. Comparison and Ablation Analysis

To quantify the improvement over our previous CNN-based system [30] and the contribution of each component, three models were trained using the same dataset, participant-level split, preprocessing procedure, and input channels, varying only the architecture: the previous CNN-based model, which performed scalar regression of peak KAM and KAM impulse; the 1D U-Net without the GRU bottleneck; and the full 1D U-Net with the GRU bottleneck. These models were evaluated using the same internal validation dataset and the evaluation metrics described above.

## 3. Results

### 3.1. Estimation Accuracy for Peak KAM and KAM Impulse

Table 2 summarizes the performance of the final IMU-based 1D U-Net model for estimating peak KAM and KAM impulse in the internal validation and independent holdout datasets, with 95% confidence intervals provided for all metrics. Absolute errors in the holdout dataset were similar to those in the internal validation dataset for both targets. The two error distributions did not differ for KAM impulse (Mann–Whitney U test, *p* = 0.561). For peak KAM, the holdout error was marginally lower (*p* = 0.049; MAE, 0.054 vs. 0.056; median, 0.041 vs. 0.048), indicating that performance did not degrade in the holdout dataset. Because this test pooled individual gait trials without accounting for within-participant correlation, and because the participant-level confidence intervals overlapped substantially, the error magnitudes were interpreted as comparable across datasets.

**Figure 3:**
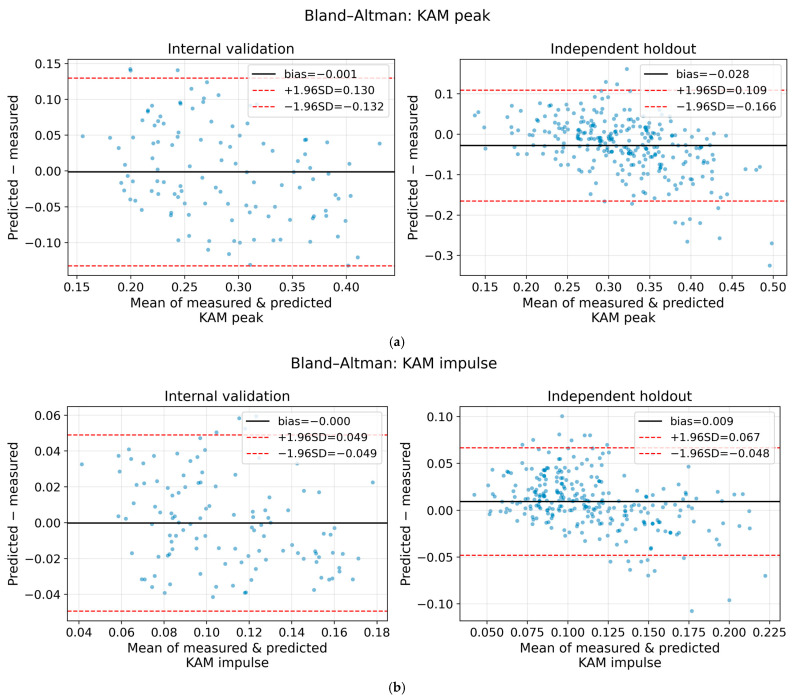
Bland–Altman plots for peak KAM and KAM impulse in the internal validation and independent holdout datasets. (**a**) Peak KAM; (**b**) KAM impulse. The solid black line indicates the mean bias, and the red dashed lines indicate the 95% limits of agreement. The vertical axis represents the difference between the predicted and reference values, and the horizontal axis represents the mean of the predicted and reference values. Units are N·m/(BW·Ht) for peak KAM and N·m·s/(BW·Ht) for KAM impulse.

Bland–Altman analyses were additionally performed to evaluate agreement between the predicted and reference values (Figure 3). For peak KAM, the bias was negligible in the internal validation dataset (approximately −0.001), whereas mild underestimation was observed in the holdout dataset (−0.028), with 95% limits of agreement of approximately ±0.13 in the internal validation dataset and −0.17 to 0.11 in the holdout dataset. For KAM impulse, the bias was also small in both datasets, with 95% limits of agreement of approximately ±0.05 in the internal validation dataset and −0.05 to 0.07 in the holdout dataset.

**Figure 4:**
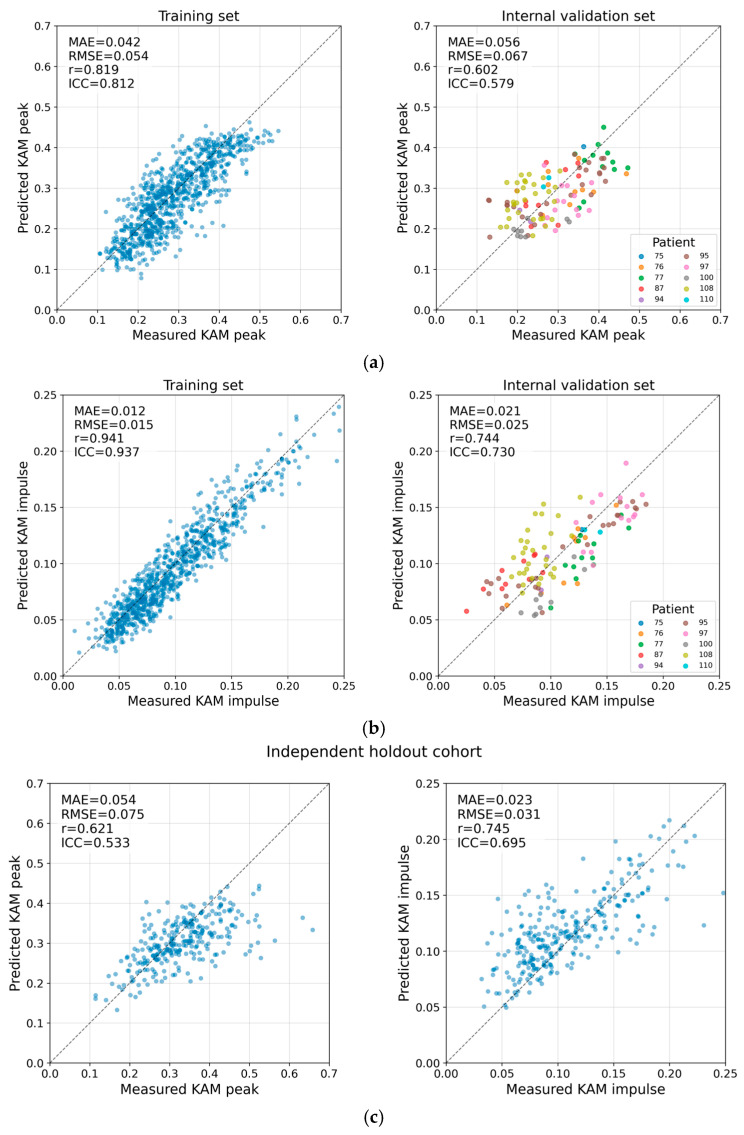
Scatter plots comparing predicted and reference values for peak KAM and KAM impulse. (**a**) Peak KAM values in the training and internal validation datasets; (**b**) KAM impulse values in the training and internal validation datasets; (**c**) Peak KAM and KAM impulse values in the independent holdout dataset. Units are N·m/(BW·Ht) for peak KAM and N·m·s/(BW·Ht) for KAM impulse.

Figure 4 presents scatter plots comparing the predicted and reference values for peak KAM and KAM impulse in the training, internal validation, and independent holdout datasets. In the training dataset, the model showed lower errors and stronger association and agreement; the MAE, RMSE, Pearson’s r, and ICC values were 0.042, 0.054, 0.819, and 0.812 for peak KAM, respectively, and 0.012, 0.015, 0.941, and 0.937 for KAM impulse, respectively. In both the internal validation and holdout datasets, the predicted values showed positive associations with the reference values, although the data points showed greater scatter than those in the training dataset. In the internal validation dataset, waveform reconstruction showed a mean range-normalized RMSE of 0.183 and a mean DTW distance of 0.260, with a waveform-level Pearson correlation coefficient of 0.898 between the predicted and reference KAM waveforms.

In participant-level 5-fold cross-validation, model performance was generally consistent across folds (Table 3). The Pearson’s r values were 0.517 ± 0.150 for peak KAM and 0.721 ± 0.060 for KAM impulse, and the values reported above fell within this cross-validation distribution.

### 3.2. Direct Comparison with the Previous CNN Model and Ablation Analysis

In the internal validation dataset, the proposed 1D U-Net with the GRU bottleneck outperformed the previous CNN-based model across all metrics (Table 4). For peak KAM, the proposed model showed higher correlation and agreement than the previous CNN-based model (r = 0.602 vs. 0.346; ICC = 0.579 vs. 0.317), whereas the difference in MAE was relatively modest (0.056 vs. 0.066). For KAM impulse, the proposed model also showed better performance than the previous CNN-based model (r = 0.744 vs. 0.383; ICC = 0.730 vs. 0.325; MAE = 0.021 vs. 0.030). Removing the GRU bottleneck reduced performance to approximately the level of the previous CNN-based model, indicating that the recurrent bottleneck was the principal contributor to the improvement.

### 3.3. Representative KAM Waveform Reconstruction

**Figure 5:**
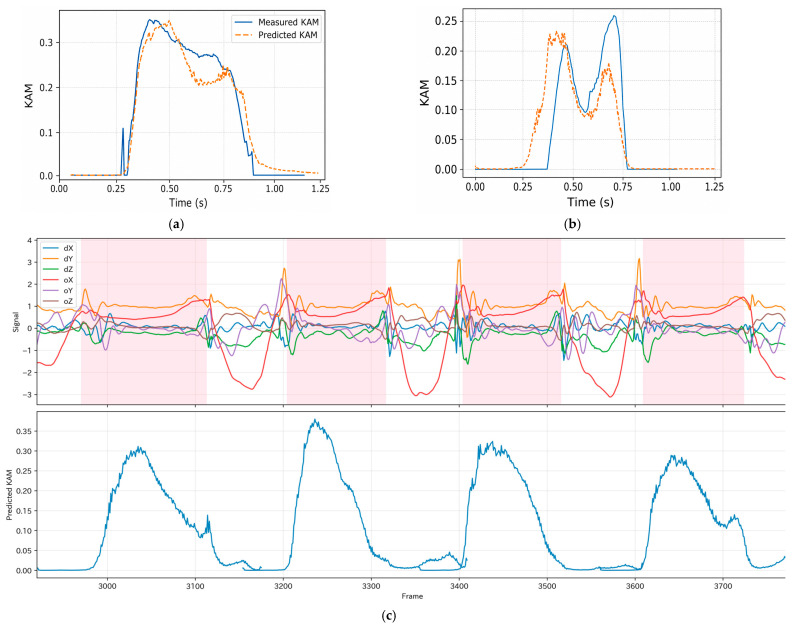
Representative waveform examples of KAM estimation by the proposed model. (**a**) An example of a gait cycle showing high agreement between the predicted and reference KAM waveforms. (**b**) An example showing a slight temporal offset at stance initiation between the predicted and reference KAM waveforms. (**c**) An example of continuous KAM waveform reconstruction during several consecutive gait cycles in a representative participant. In panels (**a**,**b**), the blue line indicates the reference KAM and the orange line indicates the predicted KAM. In panel (**c**), the upper panel shows the six-axis IMU signals used as input to the model, and the lower panel shows the reconstructed continuous KAM waveform. dX–dZ represent triaxial acceleration signals, and oX–oZ represent triaxial angular velocity signals. In the KAM waveform panels, values are expressed as N·m/(BW·Ht), and the horizontal axis represents frame number.

Figure 5 shows representative waveform comparisons between the predicted and reference KAM waveforms. In some cases, the predicted waveform closely matched the reference waveform throughout the stance phase (Figure 5a). In other cases, a slight temporal offset was observed at stance initiation between the predicted and reference signals (Figure 5b). Such offsets may be influenced, in part, by minor uncertainties in temporal alignment between the IMU signals and reference KAM waveforms during preprocessing. The model was also able to reconstruct continuous KAM waveforms across several consecutive gait cycles (Figure 5c).

## 4. Discussion

The principal findings of this study were that a single IMU attached to the proximal tibia, combined with a 1D U-Net incorporating a GRU bottleneck, enabled reconstruction of the KAM waveform during walking and that this waveform-reconstruction approach was also feasible in an independent holdout dataset. In addition, the direct comparison with the previous CNN-based model and the ablation analysis showed that the proposed model improved correlation and agreement, suggesting that the GRU bottleneck contributed to this improvement. The model was evaluated not only using correlation metrics but also using agreement analyses, which indicated small systematic biases while also showing the remaining variability of individual prediction errors. These findings are important because KAM is meaningful not only when summarized as clinically informative scalar metrics, such as peak KAM and KAM impulse, which remain widely used [12,36], but also as a time-varying biomechanical waveform reflecting medial knee joint loading throughout the stance phase [39,40]. Therefore, reconstructing the KAM waveform and subsequently deriving clinically relevant parameters, such as peak KAM and KAM impulse, from the reconstructed waveform may provide a more consistent framework than estimating these parameters as separate outputs. In this respect, the present study demonstrates that waveform-based KAM assessment is feasible using a single tibial IMU alone.

One possible explanation for the improved performance of the present system is that the 1D U-Net with a GRU bottleneck is well suited to modeling temporal structure. Its encoder–decoder structure enables integration of features across broader temporal scales, while skip connections allow local information to be preserved and incorporated into the output. In addition, the GRU bottleneck may have helped capture temporal dependencies within the compressed gait-signal representation, thereby improving the reconstruction of continuous KAM patterns. These characteristics may have been advantageous for estimating dynamic KAM patterns during stance.

The present study builds upon our previously reported KAM estimation system [30]. In the previous version, a 1D CNN was used to estimate peak KAM and KAM impulse as separate scalar outputs. Although that earlier system demonstrated the feasibility of KAM assessment, there remained room for improvement, particularly in the correlations for peak KAM and KAM impulse in the internal validation dataset. To clarify whether the present framework improved upon that previous system, we performed a direct same-data comparison and ablation analysis using the same dataset, participant-level split, preprocessing, and input channels. In this comparison, the proposed 1D U-Net with a GRU bottleneck outperformed the previous CNN-based model across all evaluated metrics, with particularly clear improvements in correlation and agreement for peak KAM and KAM impulse. For example, Pearson’s r improved from 0.346 to 0.602 for peak KAM and from 0.383 to 0.744 for KAM impulse, with corresponding improvements also observed in ICC. In contrast, the improvement in peak KAM MAE was relatively modest, from 0.066 to 0.056. Moreover, removing the GRU bottleneck reduced the performance of the U-Net model to approximately the level of the previous CNN-based model, suggesting that the recurrent bottleneck was a principal contributor to the improved temporal modeling. Thus, the present findings support the shift from separate scalar prediction toward waveform reconstruction. The main improvements were reflected in correlation, agreement, and KAM impulse estimation rather than in peak KAM absolute error alone.

Various approaches have been explored to simplify out-of-laboratory estimation of KAM or related gait kinetics, including force plate-based machine learning [28], wearable inertial sensors [20,21,22,23,24,29], instrumented insoles [41], video-based motion analysis [27,42,43], and multimodal sensor fusion [25,26]. Early wearable or inertial motion capture-based studies demonstrated the feasibility of estimating joint moments and ground reaction forces outside highly controlled laboratory settings [20,21,22,23]. However, these approaches often relied on relatively complex measurement and analysis pipelines involving multiple IMUs, plantar pressure sensors, or frameworks based on inverse dynamics and musculoskeletal modeling, which limited their practicality for simple clinical implementation. Subsequently, Wang et al. [24,44] demonstrated the feasibility of real-time KAM estimation using a multimodal sensor system and further highlighted its potential application to sensor-based gait retraining. In particular, Wang et al. reported real-time KAM estimation using data from two wearable IMU sensors, achieving R^2^ of 0.956 for an ANN model and a test R^2^ of 0.947 for an XGBoost model [24]. Feng et al. [26] also reported improved KAM estimation accuracy using a multimodal deep-learning framework integrating IMU and EMG signals. Video-based approaches have also shown promising performance; Boswell et al. [27] reported a video-oriented neural-network approach that predicted peak KAM from motion-capture-derived anatomical landmark positions simulating video keypoints, with r^2^ = 0.78 and MAE = 0.53%BW·H for 3D inputs and r^2^ = 0.85 and MAE = 0.49%BW·H for frontal-plane inputs. In addition, OpenCap [43] estimated early-stance peak KAM from smartphone videos with r^2^ = 0.80 and an MAE of 0.30%BW·Ht using a pipeline that combines pose estimation and musculoskeletal simulation. However, direct quantitative comparison is limited because these studies differed in sensor configuration, study population, prediction target, validation strategy, and outcome normalization. At the same time, these previous studies illustrate a persistent trade-off among estimation accuracy, sensor simplicity, portability, and clinical feasibility. In this context, although the present system did not necessarily exceed these previous approaches in estimation accuracy, it provides a practical framework for KAM assessment using a simpler sensor configuration. It also supports the derivation of clinically relevant parameters from the reconstructed KAM waveform.

The clinical interpretation of the present error magnitude should consider the intrinsic variability of KAM measurements. Even conventional three-dimensional gait analysis with force plates includes test–retest variability. Birmingham et al. [45] reported that, in patients with medial compartment knee osteoarthritis, the SEM and MDC95 for peak KAM were 0.36 and 1.00% BW·Ht, respectively. Tan et al. [46] similarly reported that, in knee-healthy individuals, the SEM and MDC95 for first peak KAM were 0.20 and 0.56%BW·Ht, respectively, and also showed that absolute measurement variability differed across KAM-derived measures. In the present study, the MAE for peak KAM was 0.056 N·m/(kg·m) in the internal validation dataset and 0.054 N·m/(kg·m) in the independent holdout dataset. Although SEM, MDC95, and MAE are not directly equivalent metrics, these findings suggest that the present peak KAM prediction error was larger than the SEM reported for laboratory-based measurements but was broadly comparable in magnitude to reported MDC95 values. For KAM impulse, Tan et al. reported normalized SEM values of 10–12% and MDC95 values of 27–36%, whereas the present model showed MAPE values of 23.3% and 26.8% in the internal validation and independent holdout datasets, respectively. Thus, the present prediction errors should be interpreted in the context of the inherent variability of KAM-derived measures themselves.

From an intervention perspective, gait-retraining studies generally aim to reduce KAM rather than to use estimated KAM values as standalone diagnostic thresholds. A systematic review showed that several gait modification strategies can reduce KAM, although the direction and magnitude of change vary depending on the strategy, implementation, study population, and phase of the gait cycle [11]. In a randomized controlled trial of sensor-based gait retraining in patients with early medial KOA, the first peak KAM was reduced by 16.5% after a 6-week intervention, and previous laboratory-based gait-retraining studies were reported to achieve approximately 20–22% reductions in first peak KAM [44]. Taken together, these findings indicate that the prediction error of the present model remains larger than that of laboratory-based KAM measurement and should be further reduced. However, this error should not be interpreted in isolation, but rather in relation to the inherent variability of KAM-derived measures and the magnitude of KAM reductions targeted in gait-retraining studies.

The clinical significance of this approach lies in its combination of practicality and interpretability. The system requires only a single IMU attached to the proximal tibia and does not rely on complex laboratory-based instrumentation. Therefore, it may support screening-level assessment of medial knee loading or evaluation of group-level tendencies in settings closer to routine clinical practice. In addition, because the model reconstructs the KAM waveform, it may allow the assessment of continuous mechanical information, such as loading duration and step-to-step variability, and may facilitate a more detailed characterization of individual gait patterns. Such a simple wearable assessment system may be particularly useful in patients with early KOA or in those at risk of disease progression, in whom abnormal dynamic loading may still be modifiable and early-stage evaluation and intervention may be especially valuable. At present, clinical management of KOA still depends largely on subjective symptoms such as pain, structural assessment using radiographs, and functional assessment performed in rehabilitation settings [47]. However, pain is inherently subjective, and although radiographic assessment is valuable for evaluating structural status, visible radiographic progression often becomes apparent only after structural changes have already advanced. In addition, in routine clinical practice, it is often difficult to provide sufficiently individualized gait and exercise guidance for each patient on an ongoing basis. From the perspective of both patients and clinicians, there remains a substantial need for an objective and accessible indicator that can capture knee joint mechanical loading in a simple manner. In this context, single-IMU-based KAM assessment may offer one possible approach to addressing this gap. Nevertheless, given the present error magnitude and the higher accuracy reported in some previous approaches using multiple sensors or multimodal systems, the present system should not yet be regarded as interchangeable with conventional three-dimensional gait analysis or as a standalone basis for precise individual-level clinical decision-making. Further improvements in prediction accuracy and prospective studies defining clinically meaningful change thresholds for IMU-derived KAM estimates will be necessary before this system can be used to guide individual treatment decisions or monitor small longitudinal changes.

The present study has several limitations. First, the study population was imbalanced, with fewer patients with advanced KOA than healthy individuals or patients with early KOA. Although the present system is primarily intended for preventive or early intervention strategies in populations including healthy individuals and patients with early KOA, the performance of the model in more severe cases was not sufficiently evaluated. Therefore, caution is needed when generalizing the present findings to patients with advanced KOA, and future studies including a larger number of patients with KL grade 3–4 are required to clarify model performance in this population. Second, all participants in this study were Japanese, and the generalizability of the model to populations with different ethnic and anthropometric backgrounds remains to be established. Third, although the holdout dataset was independent from the training and internal validation datasets, all data were collected within the same institutional and measurement environment under the same measurement protocol. Therefore, the present findings should be interpreted as an assessment within a single-center measurement environment. Future studies involving independent datasets collected across different institutions and clinical settings are needed to further establish the generalizability of the proposed model. Fourth, although the data were split at the participant level and confidence intervals were estimated using a participant-level cluster bootstrap, the number of independent participants available for validation was limited. Participant-level 5-fold cross-validation was additionally performed to assess robustness to the data split; however, full leave-subject-out cross-validation analysis was not performed in the present study. Further validation using larger participant-level datasets, including leave-subject-out and multicenter validation frameworks, will be necessary to confirm the robustness and generalizability of the model. Finally, as discussed above, the present system should be regarded as a simplified screening or monitoring tool rather than a substitute for conventional gait analysis, and further refinement of the model is necessary to improve its applicability to precise individual-level clinical decisions.

## 5. Conclusions

In this study, we demonstrated that a simplified wearable system combining a single tibial IMU with a 1D U-Net incorporating a GRU bottleneck could reconstruct the KAM waveform during walking, and that this waveform-reconstruction approach was also feasible in an independent holdout dataset. In addition, peak KAM and KAM impulse could be derived consistently from the reconstructed waveform, and direct comparison with the previous CNN-based model, together with ablation analysis, suggested that the GRU bottleneck contributed to improved correlation and agreement. Although further improvement in prediction accuracy is required and validation in more diverse populations and measurement environments remains necessary, the present framework may provide a practical basis for simplified screening or monitoring of medial knee loading in settings closer to routine clinical practice.

## Figures and Tables

**Table 1:** Participant characteristics of the training, internal validation, and holdout datasets.

Dataset	Participants (n)	Knees (n)	Gait trials (n)	Age (Years)	Sex (M/F)	BMI (kg/m^2^)	KL0	KL1	KL2	KL3	KL4
Training	40	78	837	63.7 ± 11.0	14/26	23.7 ± 4.2	18	23	28	9	0
Internal validation	10	17	114	64.4 ± 8.2	4/6	25.9 ± 3.7	0	2	12	2	1
Holdout	45	88	281	61.0 ± 13.6	8/37	22.3 ± 3.6	27	25	17	17	2

Values are presented as mean ± standard deviation unless otherwise indicated. KL: Kellgren–Lawrence grade. BMI: body mass index. Multiple gait trials were collected from participants, and analyses were performed at the knee level.

**Table 2:** Performance of the IMU-based 1D U-Net model for estimating peak KAM and KAM impulse in the internal validation and holdout datasets.

Metric	Peak KAM		KAM Impulse	
	Internal Validation	Holdout	Internal Validation	Holdout
MAE	0.056 [0.048–0.061]	0.054 [0.046–0.064]	0.021 [0.018–0.023]	0.023 [0.018–0.028]
RMSE	0.067 [0.060–0.070]	0.075 [0.063–0.088]	0.025 [0.021–0.028]	0.031 [0.025–0.037]
MAPE	22.2 [15.9–26.1]	15.9 [13.8–18.3]	23.3 [16.9–29.3]	26.8 [19.3–36.5]
Pearson r	0.602 [0.293–0.750]	0.621 [0.462–0.738]	0.744 [0.470–0.887]	0.745 [0.612–0.831]
Spearman *ρ*	0.578 [0.260–0.736]	0.618 [0.456–0.745]	0.718 [0.425–0.875]	0.710 [0.540–0.831]
ICC (2,1)	0.579 [0.280–0.710]	0.533 [0.370–0.659]	0.730 [0.441–0.844]	0.696 [0.529–0.803]

Values are presented as point estimates [95% confidence intervals]. MAE: mean absolute error; RMSE: root mean square error; MAPE: mean absolute percentage error; r: Pearson’s correlation coefficient; *ρ*: Spearman’s rank correlation coefficient; ICC: intraclass correlation coefficient. MAE and RMSE are expressed in N·m/(BW·Ht) for peak KAM and N·m·s/(BW·Ht) for KAM impulse; MAPE is expressed as a percentage. Pearson r and ICC are unitless. Confidence intervals were estimated using participant-level cluster bootstraps.

**Table 3:** Participant-level 5-fold cross-validation performance of the IMU-based 1D U-Net model for estimating peak KAM and KAM impulse within the model-development dataset.

Metric	Peak KAM	KAM Impulse
MAE	0.061 ± 0.008	0.023 ± 0.004
RMSE	0.076 ± 0.010	0.028 ± 0.004
Pearson r	0.517 ± 0.150	0.721 ± 0.060
ICC	0.464 ± 0.152	0.688 ± 0.052

Values are presented as mean ± standard deviation across folds. MAE: mean absolute error; RMSE: root-mean-square error; ICC: intraclass correlation coefficient. MAE and RMSE are expressed in N·m/(BW·Ht) for peak KAM and N·m·s/(BW·Ht) for KAM impulse. Pearson’s r and ICC are unitless.

**Table 4:** Direct comparison with the previous CNN-based model and ablation analysis in the internal validation dataset.

Model	Peak KAM				KAM Impulse			
	MAE	RMSE	r	ICC	MAE	RMSE	r	ICC
Previous CNN-based model	0.066	0.081	0.346	0.317	0.030	0.037	0.383	0.325
1D U-Net without GRU bottleneck	0.070	0.086	0.261	0.241	0.031	0.036	0.490	0.482
Proposed 1D U-Net with GRU bottleneck	0.056	0.067	0.602	0.579	0.021	0.025	0.744	0.730

MAE: mean absolute error; RMSE: root-mean-square error; r: Pearson’s correlation coefficient; ICC: intraclass correlation coefficient. MAE and RMSE are expressed in N·m/(BW·Ht) for peak KAM and N·m·s/(BW·Ht) for KAM impulse. r and ICC are unitless.

## Data Availability

The data presented in this study are available from the corresponding author upon reasonable request. The data are not publicly available due to privacy and ethical restrictions related to human participant data.

## Supplementary Materials

The following supporting information can be downloaded at: https://www.mdpi.com/article/10.3390/s26144421/s1, Table S1: Channel ablation analysis comparing 3-channel, 5-channel, and 6-channel input configurations on the internal validation set.
