# Supplementary material for: A Single-IMU Wearable System with 1D U-Net for Knee Adduction Moment Waveform Reconstruction During Gait
Source: Sensors (Basel). 2026 Jul 12;26(14):4421. doi: 10.3390/s26144421 (PMC13416722; doi:10.3390/s26144421)
Supplement: Supplementary file 1 [file sensors-26-04421-s001.zip › sensors-4388067-supplementary.pdf]

Table S1. Channel ablation analysis comparing 3-channel, 5-channel, and 6-channel input configurations on the internal validation dataset.

Input configuration	Peak KAM MAE	Peak KAM r	Peak KAM ICC	KAM impulse r	KAM impulse ICC
3 channels (acceleration only)	0.061	0.518	0.445	0.692	0.675
5 channels (final model; excluding x-axis angular velocity)	0.053	0.633	0.615	0.795	0.785
6 channels (including x-axis angular velocity)	0.073	0.196	0.174	0.584	0.551

All models were trained under identical conditions for the channel ablation experiment. The 5-channel configuration was used as the final input configuration. The x-axis angular velocity was used for gait-event detection during preprocessing but was excluded from the model input.
